# Virgil as a Veterinary Poet

**Published:** 1882-10

**Authors:** 


### VIRGIL AS A VETERINARY POET.

VIRGIL'S DESCRIPTION OF THE DEATH OF AN OX.—The genius of Virgil did not disdain humble themes. In the Georgics he gives faithful descriptions of rural life, descriptions that are often full of poetic beauty. He becomes here the poet of the farmer, and in some places we might add of the veterinarian. The common diseases and accidents of the sheep, cattle and horses were evidently well known to him, and some are quite fully described, "Morborum qouque causas et signa." One passage in particular (Book III. 478-515) describes the death of a ploughman's ox, as critics have affirmed, from anthrax, though the diagnosis is not so easy.

A fatal murrain has swept over the land, killing both wild and domestic animals.

"Sarpe in honore Deûm medio stans hostia ad aram
Lanea dum nivea circumdatur infula vitta,
Inter candantes cecedit moribunda ministros.

Ecce autem duro fumans sub vomere taurus
Concidit, et mixtum spumis vomit ore cruorem
Extremosque ciet genutus, it tristes arator," etc.

Which Dryden translates as follows:

"The victim ox, that was for altars prest,
Trimmed with white ribbons and with garlands drest,
Sank of himself, without the gods' command,
Preventing the slow sacrificer's hand."

And again:

"The bull, which to the yoke was bred to bow
(Studious of tillage and the crooked plough),
Falls down and dies; and dying, spews a flood
Of foamy madness, mixed with clotted blood."

In the same connection he describes most vividly the effects of the disease upon horses. The clinical symptoms resemble more those of glanders.

Possibly a reading of Virgilius Maro might improve the veterinary student's professional knowledge as well as his Latinity.

